# Single Neuroform Atlas stent: a reliable approach for treating complex wide-neck bifurcated aneurysms

**DOI:** 10.3389/fneur.2024.1391799

**Published:** 2024-07-16

**Authors:** Hong Suk Ahn, Hong Jun Jeon, Byung Moon Cho, Se Hyuck Park

**Affiliations:** Department of Neurosurgery, Kangdong Sacred Heart Hospital, Hallym University College of Medicine, Seoul, Republic of Korea

**Keywords:** coiling, wide-neck bifurcated cerebral aneurysm, Neuroform Atlas stent, bifurcation, aneurysm, stent

## Abstract

**Background:**

Treating wide-neck bifurcated cerebral aneurysms (WNBAs) using various techniques and new devices has shown favorable outcomes. However, endovascular coiling can be technically challenging when the aneurysm neck is incorporated into the parent vessel. Furthermore, although recent research has reported favorable outcomes of Neuroform Atlas stent (NAS)-assisted coiling, broad inclusion criteria have hampered precise evaluations of their effectiveness and safety for treating complex WNBAs. Therefore, this study evaluated whether the use of a single NAS is a safe and effective approach for treating complex WNBAs.

**Methods:**

We treated 76 complex WNBAs (unruptured, *n* = 49; ruptured, *n* = 27) using single NAS-assisted coil embolization and retrospectively analyzed the clinical and angiographic outcomes.

**Results:**

In a cohort of 68 patients (mean age, 58.3 ± 11.6 years; males *n* = 20, 29.4%; females, *n* = 48, 70.6%), 76 stents were successfully delivered to the target aneurysms, yielding a technical success rate of 98.6%. Complete occlusion was evident in 59 (77.6%) of 76 aneurysms, with neck remnants found in 16 (21.1%) and partial occlusion in 1 (1.3%). Treatment-related morbidities comprised one branch occlusion and one parenchymal hemorrhage. However, no new neurological symptoms of unruptured aneurysms were evident at discharge. The outcomes of 20 of the 27 ruptured aneurysms were favorable (Glasgow Outcome Scale scores of 4 or 5) at the final follow-up assessment (mean 12.2 [6–29] months), except for one initial subarachnoid hemorrhage. Post-treatment angiography revealed complete occlusion in 89.1%, neck remnants in 7.8%, and incomplete occlusion in 3.1% of the aneurysms. Approximately 88.2% of the patients were assessed at least once by follow-up diagnostic or magnetic resonance angiography (mean, 12.5 ± 4.3 [range, 6–29] months), with five (7.8%) minor and two (3.1%) major recurrences.

**Conclusion:**

A single NAS is safe and effective for treating WNBAs incorporated into parent vessels.

## 1 Introduction

Endovascular treatment for aneurysms has significantly evolved with the advancement of various devices, shifting the paradigm toward aneurysm management (1). This approach is favored for treating aneurysms as it is minimally invasive and effective. However, certain types of aneurysms, particularly wide-neck bifurcation aneurysms (WNBAs), present unique challenges due to their complexity. WNBAs are often characterized by unfavorable neck anatomy combined with incorporated vessels, necessitating precise and innovative treatment strategies.

Stent-assisted coil embolization techniques, introduced approximately 15 years ago have become indispensable for treating complex aneurysms (2). Despite the introduction of new devices such as flow diverters and the Woven EndoBridge (WEB), conventional stents continue to occupy a prominent position in the treatment arsenal (3). Their sustained relevance is attributed to high aneurysm occlusion rates, which renders them particularly suitable for managing WNBAs. Approval of the Neuroform EZ and EZ3 stents (Stryker Neurovascular, Kalamazoo, MI, United States) by the US Food and Drug Administration in 2002 has resulted in significant milestones in the evolution of stent technology (4).

The Neuroform Atlas stent (NAS) is a refined version of its predecessors with lower-profile delivery, which is a notable advancement (5, 6). Although the outcomes of treatment with NAS-assisted coiling have been favorable, the broad inclusion criteria in recent studies have hampered precise evaluations of its effectiveness and safety for treating complex WNBAs (7–10). Therefore, our study aimed to address this issue by detailing our experience with single NAS-assisted coil embolization and focusing exclusively on its application in the treatment of complex WNBAs.

## 2 Materials and methods

### 2.1 Patient population

In this retrospective study, we analyzed the records of complex WNBAs treated with endovascular coiling using a single NAS at our center. The data were sourced from a prospectively maintained neuro-interventional database between February 2018 and December 2022. Complex WNBAs were defined as aneurysms situated at major artery bifurcations and including a branch within the aneurysm sac. A “wide-neck” aneurysm is characterized by a neck diameter of ≥4 mm or a dome-to-neck ratio of ≤1.5. During the 5-year study period, 855 aneurysms in 658 patients were treated with endovascular coiling at our facility. Among them, 91 (10.6%) aneurysms met the criteria for complex WNBA. We treated 76 (8.9%) of these 91 aneurysms in 68 patients with a single NAS technique. Patients with aneurysms located at major artery bifurcations, including internal carotid artery bifurcation (ICBIF), middle cerebral artery bifurcation (MCBIF), anterior communicating artery (AcomA), basilar artery bifurcation (BABIF), and posterior-inferior cerebellar artery (PICA), were eligible for inclusion. The mean diameter of the included aneurysm was 8.7 mm, and the neck diameter was on average 4.1 mm. Clinical and imaging data were collected from medical charts, Picture Archiving and Communication System (PACS), and our neuro-interventional database. Informed consent was obtained before treatment. Our institutional review board approved this retrospective study (KANGDONG 2023-01-016-003) and waived the need for informed consent to study inclusion owing to the retrospective analysis of innominate data.

### 2.2 Endovascular treatment

All patients with unruptured aneurysms were administered dual antiplatelet medication (aspirin 100 mg and clopidogrel 75 mg) for a minimum of 5 days before treatment. Prior to the procedure, a platelet function test (VerifyNow assay; Accumetrics, San Diego, CA, United States) was conducted to ensure a good response to aspirin and clopidogrel. Additional doses of aspirin or clopidogrel (300 mg) were administered to patients with poor responses. An initial bolus of heparin (3,000–4,000 IU) was intravenously injected during the procedure to initiate anticoagulation followed by hourly booster doses. Patients with ruptured aneurysms were not administered pre-operative antiplatelet therapy or an intra-operative heparin bolus. Dual antiplatelet medication with aspirin and clopidogrel (300 mg each) was administered immediately after stent deployment. This post-procedural regimen was maintained for at least 6 months, then transitioned to a single antiplatelet regimen for at least 12 months.

Under general anesthesia, a 6F Envoy (Codman & Shurtleff, Raynham, MA, United States) or a 6F Benchmark (Penumbra, Inc., Alameda, CA, United States) guiding catheter was carefully placed in the relevant internal carotid artery or dominant vertebral artery. Aneurysmal morphology, including the size of parent vessels, was measured using reconstructed three-dimensional rotational angiography (3D-RA) images. Working projections were adjusted to characterize the width of the aneurysm neck and secure the incorporated branch via stent deployment. Pre-shaped or steam-shaped 0.010-inch Excelsior microcatheters (Stryker Neurovascular, Kalamazoo, MI, United States) with a Traxcess 0.014-inch micro guidewire (MicroVention, Tustin, CA, United States) were used for stent deployment and coil delivery. The NAS was predominantly used for all aneurysms because it comprises a low-profile, self-expanding, nitinol microstent, which enhances stent deliverability with less foreshortening, and its hybrid cell structure has better scaffolding and conforms to vessel walls more effectively than previous stents (5, 11). Specifically, the microcatheter is designed to pass the proximally closed line and re-cross the open cell for trans-strut insertion. Stent diameters were selected primarily as recommended by the manufacturers. However, we selected a 3-mm diameter stent when the parent vessel was <2 mm. The stent length was determined to cover the aneurysmal neck with proximal and distal parts extending at least 5 mm. We used stents with diameters and lengths of 3 × 15 or 3 × 21 mm for the MCBIF AcomA, and PICA, and 4.0 or 4.5 × 21 mm for the ICBIF and BABIF.

### 2.3 Outcome measures

The effectiveness of the procedure was immediately assessed as coil packing density and aneurysm occlusion rates according to the Raymond–Roy occlusion classification (RROC) (12). Patients were routinely followed up using high-resolution time-of-flight magnetic resonance angiography (MRA) at 6 months and digital subtraction angiography (DSA) at 1 year. Depending on MRA findings, DSA was performed to determine whether retreatment was necessary. The follow-up angiographic outcomes were classified as improved or stable, minor recurrence (aneurysm recurrence not requiring repeat treatment), and major recurrence (aneurysm recurrence with repeated treatment deemed necessary by the consensus of two interventionists). Technical complications, such as stent migration, incomplete deployment, and in-stent thrombosis, were also investigated.

Clinical outcomes were assessed using the Glasgow Outcome Scale (GOS) scores at discharge, and patients with rupture were assessed at the latest follow-up. Postoperative ischemic symptoms were classified as transient ischemic attack (TIA), minor stroke, or major stroke. Major stroke was defined as a new neurological event persisting for >24 h with an increase in National Institutes of Health Stroke Scale (NIHSS) scores >4 compared with baseline values. Minor stroke was defined as the absence of clinical consequences during the initial hospital stay or at 6 months post-procedure. We defined TIA as a minor stroke without restricted diffusion on magnetic resonance images and confirmed treatment-related complications, such as repeated bleeding of a ruptured aneurysm or thromboembolic events requiring intra-arterial thrombectomy.

### 2.4 Statistical analysis

All statistical analyses were performed using SPSS version 20 (IBM-SPSS Statistics for Windows, Armonk, NY, United States). All categorical variables are presented as percentages and 95% confidence intervals. All continuous variables are presented as a mean ± standard deviation (SD).

## 3 Results

### 3.1 Demographics of patients and aneurysms

Overall, 30 ruptured and 46 unruptured complex WNBAs identified in 68 patients (mean age, 58.3 ± 11.6; males, *n* = 20; females, *n* = 48) were treated with a single NAS. The locations of the treated aneurysms were as follows: MCBID (*n* = 36; 47.7%), AcomA (*n* = 20; 26.3%), BABIF (*n* = 10; 13.2%), ICBIF (*n* = 6; 7.9%), and PICA (*n* = 4; 5.3%). The mean aneurysm diameter was 8.7 ± 4.5 (range, 3.5–17.0) mm, and the neck diameter was 4.1 ± 2.3 (range, 3.2–13.7) mm with a mean aspect ratio of 1.06 ± 0.24 (range, 0.8–1.3). The mean diameters of the proximal and distal parent arteries were 2.9 ± 0.7 (range, 1.5–4.2) and 1.9 ± 0.4 (range, 1.2–3.4) mm, respectively. Coiling was completed with a combined multi-catheter in 64 (84.2%) cases, microcatheter jailing in 74 (97.4%) cases, trans-strut selection in 68 (89.5%) cases, and partial deployment to make a coil frame in 3 (3.9%) cases (Table 1).

**Table 1:** Baseline patient characteristics.

No. of patients (No. of aneurysms coiled)	68 (76)
Mean age, y	58.3 ± 11.6 (34–78)
Males: Females	20 (29.4%): 48 (70.6%)
Presentation with subarachnoid hemorrhage	27 (39.7%)
Sac diameter, mm	8.7 ± 4.5 (3.5–17.0)
Neck diameter, mm	4.1 ± 2.3 (3.2–13.7)
Aspect ratio	1.06 ± 0.24 (0.8–1.3)
Proximal parent artery diameter, mm	2.9 ± 0.7 (1.5–4.2)
Distal parent artery diameter, mm	1.9 ± 0.4 (1.2–3.4)
**Location of complex bifurcated aneurysm**	
Internal carotid artery bifurcation	6 (7.9%)
Middle cerebral artery bifurcation	36 (47.4%)
Anterior communicating artery	20 (26.3%)
Basilar artery bifurcation	10 (13.2%)
Posterior inferior cerebellar artery	4 (5.3%)
**Microcatheter/Coil technique**	
Microcatheter jailing	74 (97.4%)
Trans-strut selection	68 (89.5%)
Partial deployment with coil framing	3 (3.9%)
Combined multi-catheter	64 (84.2%)

Data are shown as means ± SD with ranges, or as numbers (*n*), or *n* (%). SD, standard deviation.

### 3.2 Technical results and angiographic and clinical outcomes

The NAS-assisted coil embolization yielded a 98.6% technical success rate in 75 out of 76 patients. The mean coil packing density was 42.1% ± 4.3% (range, 27–54%). Stent migration occurred in three (3.9%) cases, and in-stent thrombosis was observed in three (3.9%) cases. A distal segment of the stent that migrated into a large aneurysm with a 7.2-mm neck and the acute angle of the incorporated branch was retrieved using a 2.0-mm microsnare, and another stent was deployed without complications. In two cases, the stent partially migrated; however, the remaining anchoring part was sufficient to ensure successful coiling. Arterial and intravenous tirofiban injections managed in-stent thromboses in one of two patients. Immediate post-treatment angiography revealed complete occlusion in 59 (77.6%) patients, neck remnants in 16 (21.1%) patients, and incomplete occlusion in 1 (1.3%) patient. Follow-up angiograms were performed on 88.2% of patients (*n* = 60, 64 aneurysms: mean, 12.5 ± 4.3 months; range, 6–29 months). Only 11 (16.1%) patients underwent MRA. Aneurysmal recurrences in seven (10.9%) patients were minor in five and major in two, the latter of which were treated using simple coiling. Two (2.9%) patients with ruptured aneurysms (95% CI, 0.42–3.26%) developed treatment-related morbidities comprising branch occlusion (*n* = 1) and parenchymal hemorrhage (*n* = 1) after the administration of intra-arterial tirofiban. No mortality was treatment-related. Seven patients had ischemic symptoms, comprising four (5.3%) transient ischemic attacks, two (2.6%) minor strokes, and one (1.3%) major stroke. The patient with the major stroke presented with grade 2 side weakness that did not improve after 1 year of follow-up (Table 2). The GOS scores in 27 patients with rupture were 5, 4, 3, 2, in 16 (59.3%), 4 (14.8%), 4 (14.8%), and 2 (7.4%), respectively, and only 1 (3.7%) patient scored 1 (Table 3).

**Table 2:** Technical, angiographic, and clinical outcomes.

Technical success rate *n* (%)	75 (98.6%)
Coil packing density (%)	42.1 ± 4.3 (27–49)
**Initial device failure**	
Stent migration	3 (3.9%)
Incomplete deployment	0 (0%)
In-stent thrombosis	3 (3.9%)
**Initial occlusion class**	
Raymond 1	59 (77.6%)
Raymond 2	16 (21.1%)
Raymond 3	1 (1.3%)
Follow-up angiograms	60 (88.2%) of 64
Duration, months	12.5 ± 4.3 (6–29)
**Follow-up results**	
Improved or stable	57 (89.1%)
Minor recurrence	5 (7.8%)
Major recurrence	2 (3.1%)
Treatment-related morbidity	2 (2.9%; 95% CI, 0.42–3.26%)
Treatment-related mortality	0
**Symptomatic ischemic postoperative event**	
Transient ischemic deficit	4 (5.3%)
Minor stroke	2 (2.6%)
Major stroke	1 (1.3%)

Data are shown as means ± SD with ranges, or as *n* (%). 95% CI, 95% confidence interval. SD, standard deviation.

**Table 3:** Glasgow outcome scale scores in 27 ruptured cases.

Glasgow outcome scale scores	Number of patients (%)
5	16 (59.3%)
4	4 (14.8%)
3	4 (14.8%)
2	2 (7.4%)
1	1 (3.7%)

Data are shown as *n* (%).

### 3.3 Example cases

#### 3.3.1 Case 1: unruptured complex MCBIF aneurysm

**Figure 1:**
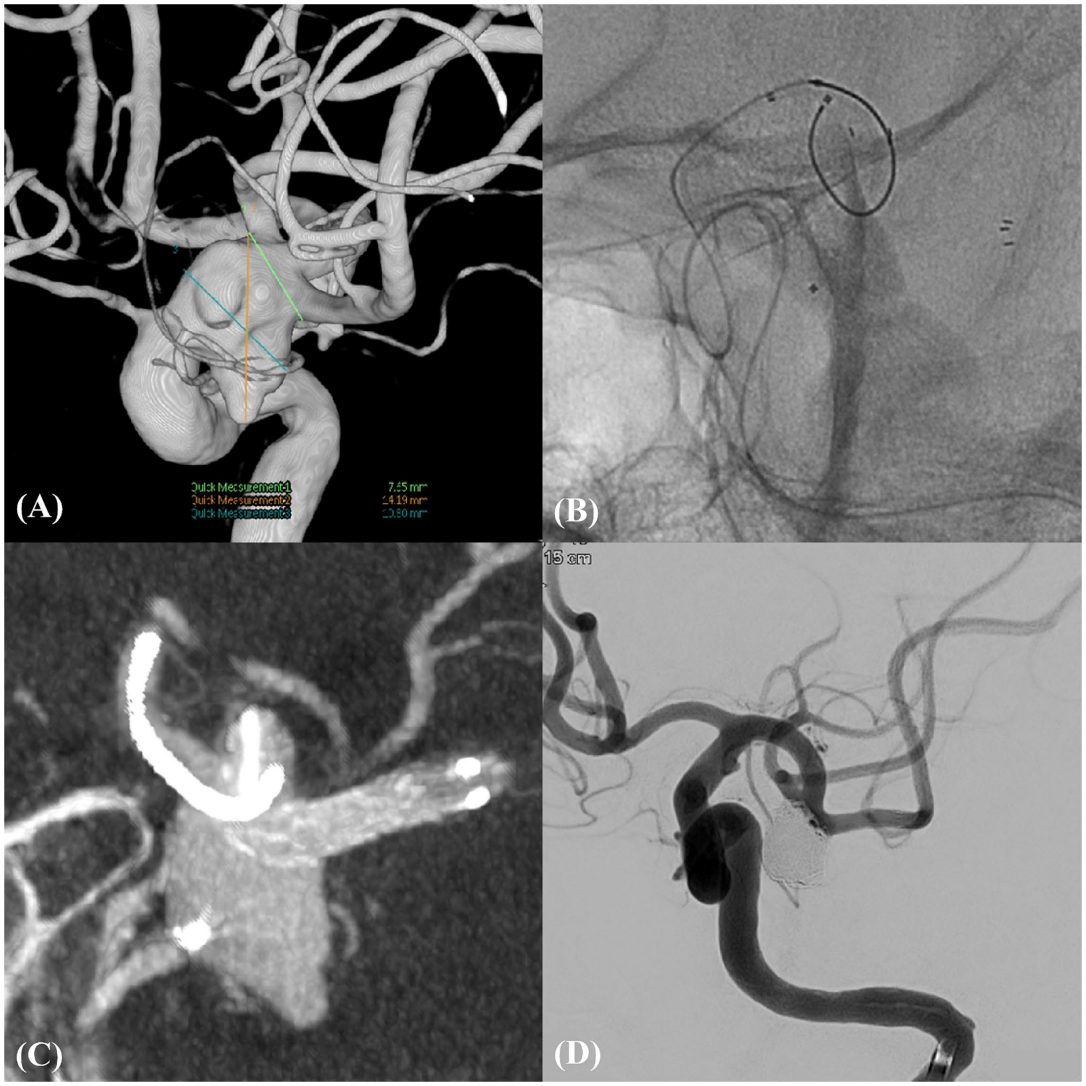
Unruptured complex aneurysm in left MCBIF (Case example 1). **(A)** An unruptured wide-neck aneurysm in the left middle cerebral bifurcation (MCBIF) encompassing both M2 branches. **(B)** Illustration of the technique of catheter protection of the M2 superior branch coupled with the strategic deployment of the neurovascular-assisted stent (NAS) spanning from the distal M1 to the M2 inferior branch. **(C)** High-resolution cone-beam computed tomography image, providing a detailed view of the microcatheter and NAS *in situ*, emphasizing the precision of device placement. **(D)** Post-procedural working angiographic view, highlighting the successfully coiled aneurysm and ensuring complete occlusion.

A 67-year-old female presented with a large, unruptured, complex aneurysm at the left MCBIF with a length and neck diameter of 24.19 and 7.65 mm, respectively. Cerebral angiography revealed the incorporation of both M2 branches into the aneurysm sac. The intervention involved deploying a 3 × 21-mm NAS from the distal M1 to the M2 inferior branch, with concurrent catheter protection of the M2 superior branch. This approach achieved complete aneurysm occlusion without complications. The MRA findings at the 2-year follow-up revealed stable occlusion with RROC III (Figure 1).

#### 3.3.2 Case 2: ruptured complex AcomA aneurysm

**Figure 2:**
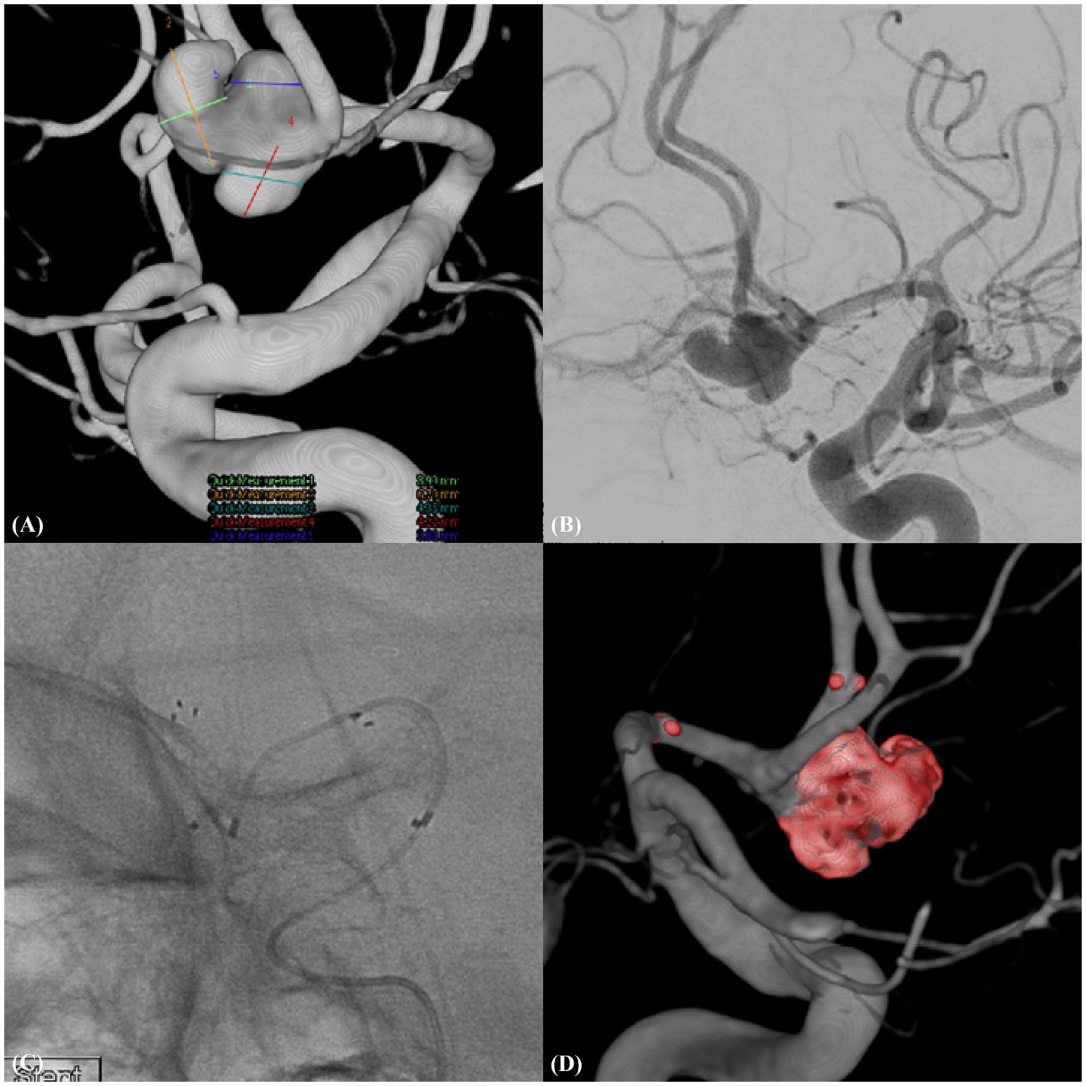
Ruptured trilobulated complex AcomA aneurysm (Case example 2). **(A)** A complex, trilobulated ruptured aneurysm of the anterior communicating artery (AcomA) with a wide neck, incorporating the left anterior cerebral artery (A1), emphasizing the challenging morphology of the aneurysm. **(B)** Detailed working view of the aneurysm illustrating its intricate morphology and relationship with the adjacent vascular structures. **(C)** Features of the deployed neurovascular-assisted stent (NAS) along with the selected microcatheter positioned for therapeutic intervention. **(D)** Post-procedural three-dimensional rotational angiogram confirming the position and effect of the treatment.

A 51-year-old male presented with a ruptured complex aneurysm of the AcomA. The width of the wide-necked, trilobulated aneurysm that incorporated with the left A1 was 8.95 mm and that of the neck was 4.72 mm. We deployed a 3.0 × 21-mm NAS from the A1 to the ipsilateral A2, resulting in complete aneurysm occlusion without complications. One-year follow-up MRA revealed stable occlusion (Figure 2).

#### 3.3.3 Case 3: recurrent left complex PICA aneurysm

**Figure 3:**
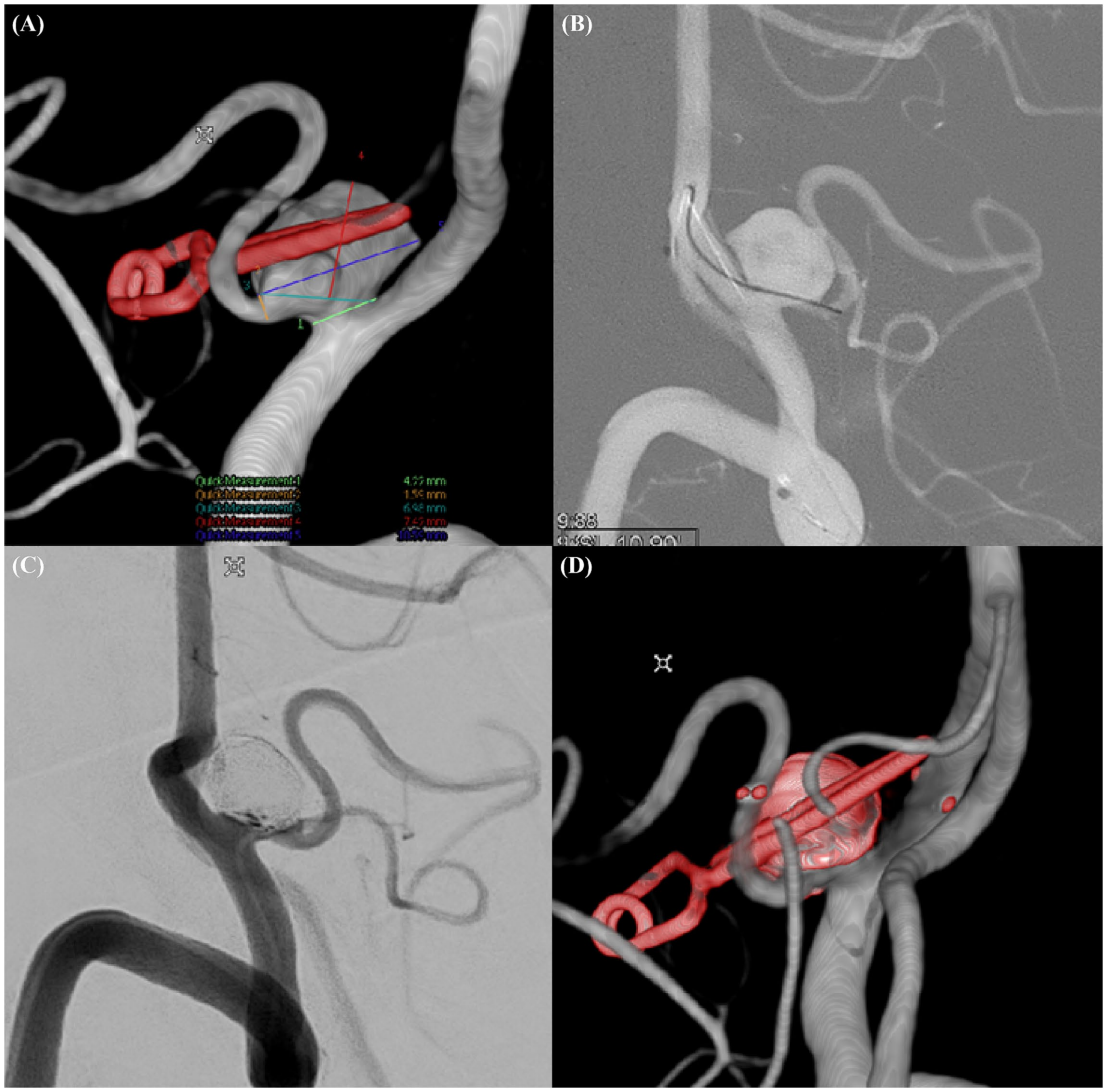
Recurrent complex aneurysm in left PICA (Case example 3). **(A)** Detailed three-dimensional rotational angiogram of a recurrent wide-necked aneurysm incorporated into the left posterior inferior cerebellar artery (PICA). **(B)** Selection of the PICA with a microwire accessed via the contralateral vertebral artery. **(C)** Postprocedural working view of the aneurysm after coiling. **(D)** Postprocedural three-dimensional rotational angiogram demonstrating the outcome of the intervention.

A 73-year-old male presented with a recurrence of a left PICA aneurysm that had been clipped 16 years earlier. Follow-up MRA and transcranial focal cerebral angiography revealed a recurrent 10.59-mm-wide aneurysm segment with a neck diameter of 6.98 mm. We accessed the PICA via the contralateral vertebral artery and deployed a 3.0 × 21-mm NAS. Despite the narrow diameter of the PICA (1.79 mm), good blood flow was maintained after NAS deployment. The aneurysm was occluded without complications and remained stable on 2-year follow-up MRA images (Figure 3).

## 4 Discussion

Endovascular coiling of complex WNBAs presents significant clinical challenges, largely attributable to their inherent anatomical complexities (13, 14). These aneurysms have wide necks and can involve several branches of the parent artery, complicating the efficacy of conventional endovascular approaches. Traditional methods, while predominant, have been associated with an increased risk of thrombosis in branching arteries, coil migration, and protrusion, highlighting the critical need for advanced treatment strategies. In response, several sophisticated techniques, such as horizontal stenting, waffle cone configuration stenting, and X- or Y-configuration double stenting, have been developed (15–19). However, these approaches require advanced technical expertise and are not devoid of additional risks.

Given these considerations, the efficacy of a single NAS is worth discussing. It features a low-profile structure coupled with superior trackability and facilitates navigation through the intricate vascular architecture that is characteristic of WNBAs. This design ensures effective deployment and secure coil retention, which has been substantiated by favorable occlusion rates in initial clinical applications (11, 20–23). Importantly, the utilization of the NAS potentially averts the need for more complex and technically demanding procedures, thereby streamlining the endovascular treatment of complex WNBAs and enhancing patient outcomes.

The innovative design of the NAS, which integrates open- and closed-cell structures, offers enhanced flexibility and adaptability to various vessel geometries. This hybrid design is pivotal for facilitating segmental opening during deployment, ensuring stable positioning and immediate apposition to the vessel wall, which is particularly advantageous for managing bifurcated aneurysms (9, 20, 24). The NAS has unique actions, such as a saddling effect and gator backing due to its segmental opening structure, which allows for a comparatively wider coverage of aneurysm necks than other remodeling stents (25). Furthermore, the low metal coverage area of the NAS (6–12%) is instrumental in achieving precise localization and minimizing post-deployment shrinkage (9). These design features are critical for maintaining the patency of the parent artery and ensuring uninterrupted blood flow post-procedure, thereby contributing to the effectiveness of the stent in the clinical setting.

The NAS has demonstrated notable efficacy in the treatment of WNBAs, as evidenced by the results of multiple clinical studies. A pivotal trial involving anterior circulation aneurysms found immediate, follow-up, and overall occlusion rates of 88, 82, and 6.2%, respectively, at 12 months, highlighting the effectiveness of the stent (7). The findings of a study of posterior circulation aneurysms showed a 100% technical success rate, with an 85.3% complete occlusion rate in the first year, which also confirmed the applicability of the NAS to complex vascular territories (5). This was corroborated by a multicenter European post-market study that found technical success and complete occlusion rates of 96.9 and 90.3%, respectively, at 12–16 months of follow-up, indicating consistent NAS performance across diverse cerebral circulations (26). A systematic review and meta-analysis reinforced these outcomes, revealing a technical success rate of 100% in 12 studies and 98% in another, with an immediate occlusion rate of 88%, which improved to 90% at 6 months and to 93% at approximately 9 months of follow-up, while maintaining a periprocedural complication rate of 5% (27). We duplicated these findings with a technical success rate of 98.6%, a mean coil packing density of approximately 42%, and Raymond grade I and II occlusions of 77.6 and 21.1%, respectively, immediately post-embolization, with a complication rate of 2.9%. At 12.5 months of follow-up, we achieved adequate occlusion in 89.1% of aneurysms. These results show the potential of NAS for adequately managing complex WNBA geometries, which is a significant stride considering the challenges often faced during neurovascular interventions.

In the evolving field of endovascular treatment for WNBAs, various techniques and devices have been explored, each with different outcomes. For instance, the waffle cone technique has shown promising immediate occlusion results with primarily mild ischemic complications, although it is limited by a lack of extensive long-term follow-up data (13, 28). Y-stenting has demonstrated occlusion rates of 47.6–95.4%, caveated with a notable complication rate of approximately 8.9% (29, 30). T-stenting, as reported by Aydin et al., achieved an 83.3% immediate occlusion rate, with occlusion increasing to 90% at follow-up. However, it is accompanied by a higher complication rate of 13.7% compared with single-stent approaches (31). Flow diversion in bifurcation aneurysms is associated with a 68% complete occlusion rate at 16 months; however, this technique bears a significant 22% complication rate that is predominantly ischemic, which has triggered debate about its clinical viability (32). Meanwhile, the WEB device has a 1-year complete occlusion rate of 52.9% and has achieved adequate occlusion in 79.1% of aneurysms, indicating promising clinical experiences; nonetheless, careful consideration for individual aneurysm shapes and vascular structures is required (33, 34). Balloon-assisted coiling involves the temporary inflation of a balloon inside the parent artery to aid in supporting and maintaining coil placement within the aneurysm sac. Although it is effective for wide-neck aneurysms and particularly useful for treating ruptured aneurysms in the acute phase when dual antiplatelet therapy is not recommended, it does not substantially enhance anatomical outcomes and can present technical challenges when using a double-balloon technique for WNBA (35, 36). We achieved success by effectively widening stent coverage at the aneurysm neck, ensuring sufficient stent length for deployment at both proximal and distal ends, and meticulously expanding the stent in the neck region to provide stable support for the primary frame coil. Consequently, the use of a single NAS for the treatment of complex WNBAs can be considered efficient and safe, as evidenced by our clinical outcomes.

The present study focused on the endovascular treatment of complex WNBAs in 68 patients and identified distinct outcomes between patients with ruptured and unruptured aneurysms. Among 41 patients with unruptured aneurysms, only 2 (4.8%) developed minor ischemic symptoms attributable to stent-related thromboembolism. Conversely, in the group of 27 patients with ruptured aneurysms, 2 (7.8%) patients developed severe complications, namely in-stent thrombosis leading to occlusion of the superior branch of the M2 artery and post-interventional parenchymal hemorrhage after intra-arterial tirofiban injection, culminating in permanent disability. The increased risk in patients with ruptured aneurysms might be attributed to the lack of premedication with antithrombotic agents and the inherently hypercoagulable state associated with their condition (37). A review of the literature indicates that the clinical outcomes of stent-assisted coiling are generally favorable in 63–94% of patients, with thromboembolic and hemorrhagic complications found in 4–17% and 1–8% of procedures, respectively (38–40). Notably, studies focusing solely on the use of the NAS to treat wide-neck aneurysms reported an overall stroke rate of 7.1%, with ischemic stroke comprising 4.7% of these incidents (27, 41). Our study observed that all seven patients with clinically unfavorable outcomes had ruptured aneurysms. Five of them developed complications due to the initial subarachnoid hemorrhage insult, which is not found in patients with unruptured aneurysms. Overall, the outcomes for 61 (89.7%) of 68 patients at the 12-month follow-up were favorable. This result not only aligns with but also surpasses the general success rates of stent treatments and is comparable to other studies involving the NAS.

Although this study provides valuable insights into the use of a single NAS for treating complex WNBA, it is important to acknowledge the limitations inherent to its retrospective design. Such limitations necessitate a cautious interpretation of our findings. Further investigation is essential for a more comprehensive understanding of the role of the NAS in this context, and prospective randomized controlled trials are required to confirm our findings. Future studies should compare the outcomes of a single NAS with other similar devices or techniques. This approach is crucial for establishing long-term effectiveness and safety profiles of the NAS for treating complex WNBAs.

## 5 Conclusion

Deployment of a single NFA stent has considerable promise for treating complex WNBAs. Its low profile and segmental opening design enable wider coverage of the aneurysm neck while preserving vital adjacent branches, thus contributing to high occlusion and low complication rates.

## Data availability statement

The raw data supporting the conclusions of this article will be made available by the authors, without undue reservation.

## Ethics statement

The studies involving humans were approved by KANGDONG institutional review board. The studies were conducted in accordance with the local legislation and institutional requirements. Written informed consent for participation was not required from the participants or the participants’ legal guardians/next of kin in accordance with the national legislation and institutional requirements. Written informed consent was obtained from the individual(s) for the publication of any potentially identifiable images or data included in this article.

## Author contributions

HA: Writing – review & editing, Writing – original draft, Visualization, Validation, Software, Project administration, Methodology, Investigation, Formal analysis, Conceptualization. HJ: Writing – review & editing, Software, Funding acquisition, Data curation. BC: Writing – review & editing, Supervision, Resources, Data curation. SP: Writing – review & editing, Supervision, Resources.
